# Do I Look Gawky? The Association between Pubertal Asynchrony and Peer Victimization

**DOI:** 10.3390/children8090794

**Published:** 2021-09-10

**Authors:** Misaki N. Natsuaki, Sofia T. Stepanyan, Jenae M. Neiderhiser, Daniel S. Shaw, Jody M. Ganiban, David Reiss, Leslie D. Leve

**Affiliations:** 1 Department of Psychology, University of California, Riverside, CA 92521, USA; 2 Department of Psychology, Gannon University, Erie, PA 16501, USA; stepanya003@gannon.edu; 3 Department of Psychology, The Pennsylvania State University, University Park, PA 16802, USA; jenaemn@psu.edu; 4 Department of Psychology, University of Pittsburgh, Pittsburgh, PA 15260, USA; danielshaw@pitt.edu; 5 Department of Psychological and Brain Sciences, The George Washington University, Washington, DC 20052, USA; ganiban@gwu.edu; 6 Yale Child Study Center, Yale University, New Haven, CT 06520, USA; reiss@yale.edu; 7 Prevention Science Institute, University of Oregon, Eugene, OR 97403, USA; leve@uoregon.edu

**Keywords:** puberty, pubertal asynchrony, peer victimization, sex differences

## Abstract

Pubertal synchrony is defined as the degree of coherence to which puberty-related body changes (e.g., breast development, growth spurt, voice change, underarm hair growth) are coordinated. During the pubertal transition, youth’s body parts grow asynchronously, making each youth’s physical appearance unique. Physical appearance is a known correlate of youth’s psychosocial functioning during adolescence, but we know little about how pubertal asynchrony plays a role in their peer relationships. Using data from an adoption study (the Early Growth and Development Study; *n* = 413; 237 boys, 176 girls), this study examined the effect of pubertal asynchrony on peer victimization. Results revealed sex-specific effects of pubertal asynchrony; pubertal asynchrony was associated with a higher risk of peer victimization for girls but a lower risk for boys. Findings highlight the intersection of physical development and social context in understanding youth’s experiences of puberty.

## 1. Introduction

The literature on puberty and its effect on psychosocial outcomes has conceptualized puberty in one of the three ways: pubertal timing, status, or tempo [1]. *Pubertal timing*—the most widely studied of the three constructs concerning psychopathology–refers to the time at which individuals’ puberty-related physical milestones occur relative to their same-age, same-sex peers. On the other hand, *pubertal status* refers to the progression of biological maturation in each child’s maturation timetable. Lastly, *pubertal tempo*—an old concept that has recently received revitalized attention–refers to how quickly (or slowly) one progresses through the stages of puberty. Research has shown that early pubertal maturation and a quick pubertal tempo (i.e., shorter duration from the start to the completion of pubertal transition) put youth, especially girls, at a heightened risk for psychological difficulties, perhaps because an abrupt and untimely biological change demands a swift adaptation to the new body and the associated social expectations [2,3]. Although the research on pubertal timing, status, and tempo has become substantively and methodologically sophisticated, there is a fourth dimension of puberty that has been understudied: *pubertal synchrony*.

### 1.1. Pubertal Synchrony

Puberty synchrony is defined as the degree of coordination among the growth of different body parts in an individual during the pubertal transition [1]. Pubertal maturation encompasses a series of sex-specific (e.g., breasts for girls, facial hair for boys) and sex-general (e.g., growth spurt, skin change) changes. These developments are coordinated by underlying biological mechanisms. As expected, the growth of various body parts reaches synchrony at the beginning and at the end of the pubertal transition. However, some degree of pubertal asynchrony is expected during the transition because each body part develops at a different time and pace (Dorn et al., 2006). Typically, girls experience breast budding and signs of a growth spurt followed by the appearance of pubic and underarm hair, then menarche [4]. Boys typically experience the pubertal transition in the following sequence: the growth of testes usually occurs first, then the appearance of pubic hair, a growth spurt, and the growth of the penis follow within a few months. In addition, increasing levels of androgen that underly many of puberty-related body changes also lead to additional changes such as oiliness in skin and hair, acne, and body odor [4], which greatly vary by individuals in terms of whether, when, and how they appear. 

However, these sequences of pubertal events reflect only general guidelines; in reality, body parts change more flexibly and variably. In a classic study of puberty among girls, Marshall and Tanner [5] observed that the stages of public hair and breast development were often incongruent within each girl’s body. For instance, among girls who had reached Tanner Stage 2 for pubic hair, 16% were at Stage 1 for breast development, 49% were at Stage 2, 27% were at Stage 3, and 8% were at Stage 4. Similarly, among girls who were at Tanner Stage 2 for breast development, 61% were at Stage 1 for pubic hair, 29% were at Stage 2, 8% were at Stage 3, and 2% were at Stage 4. More recently, Biro and colleagues [6] categorized girls into three groups based on their first sign of puberty: those with thelarche (breast development first), those with pubarche (pubic hair first), and those who experience both at the same time. For boys, a similar degree of individual differences in the temporal variation in the growth of body parts has been reported. Among boys at Tanner Stage 2 for pubic hair, 1% were at Stage 1 for penis growth, 13% were at Stage 2, 45% were at Stage 3, and 41% were at Stage 4 [7]. Extreme asynchrony may be observed in cases of precocious puberty, such as premature thelarche (premature appearance of breast development as early as the first 3–4 years of life that does not accompany other puberty-related growth) [4].

Although we do not know the source of individual variability in pubertal synchrony [8], the normative individual differences observed in pubertal synchrony would likely make the physical appearance of each youth’s body unique during the physical transformation. This study investigates whether and how individual differences in pubertal synchrony exert an impact on the social world of youth with respect to peer victimization.

### 1.2. Pubertal Asynchrony and Peer Victimization

As pubertal asynchrony could temporarily lead to a gawky physical appearance (e.g., an awkward look created by the mismatch between various body parts with differing levels of maturity), one would expect asynchrony to be studied in conjunction with psychosocial development. However, studies of pubertal synchrony are surprisingly rare, and none that we are aware of has studied the social impact of pubertal synchrony on child development. Mendle [1] provided two competing hypotheses regarding the direction of the synchrony effect. One hypothesis is that *synchronous puberty* may lead to higher rates of psychosocial maladjustment; youth who undergo coordinated maturation may find the transition doubly difficult because multiple simultaneous changes are overwhelming. The counterhypothesis is that *greater asynchrony* leads to maladjustment because asynchrony makes the pubertal transition unpredictable and leads to an imbalanced and gawky body shape at times. 

While Mendle’s [1] propositions concern adolescent mental health implications, it is quite plausible to apply them to social development and suspect that the awkwardness in physical appearance created by pubertal asynchrony could make it more difficult for youth to navigate their social world. For example, consider an asynchronously maturing boy who has reached the height of an adult man but still has a child-like high-pitched voice. Alternatively, imagine a girl who otherwise looks like a prepubertal teen except that she has growing breasts that appear to be disproportionately developed compared to the rest of her body. Such discordance in the development of different body parts often becomes a target of peer harassment. Indeed, research on adolescent peer relationships acknowledges that puberty, which signifies sexual maturation to the outer world, provides new motivations and reasons to embarrass and harass one another [9,10]. Early puberty is identified as a risk for peer victimization [11,12], especially sexual and social harassment [9,13,14,15] in early adolescence [16], but the evidence is somewhat mixed for boys with some studies identifying late maturers at heightened risk of becoming victims of peer harassment [17]. To date, there are no studies on how pubertal asynchrony affects peer victimization.

There are only two studies that we are aware of that empirically tested the effect of pubertal asynchrony on psychosocial adjustment, both of which focused on depressive symptoms. Thompson and colleagues [18] identified three groups of pubertal asynchrony patterns in a study of 13-year old girls: girls whose breast and pubic hair growth were synchronous (i.e., synchrony group), girls whose Tanner stage rating for pubic hair was greater than their Tanner stage rating for breast development (i.e., pubarche group), and girls whose Tanner stage rating for breast development was greater than their Tanner stage rating for pubic hair growth (i.e., thelarche group). The results showed that the synchronous group had fewer depressive symptoms at age 20 than the thelarche or pubarche groups, but no such association was found at age 15. In a shorter-term longitudinal study, Stumper and colleagues [19] found that compared to their peers, girls with asynchronous development (i.e., larger discrepancy between self-reported breast development and body hair growth) at Time 1 had higher levels of depression at Time 2. For boys, this association was reversed; boys with asynchronous development (i.e., larger discrepancy between self-reported voice change and body hair growth) had lower levels of depressive symptoms than their counterparts. These findings suggest that pubertal asynchrony may serve as a risk for depressive symptoms for girls but a protective factor for boys. Race did not qualify this sex-specific effect. 

This initial empirical evidence supporting the role of pubertal asynchrony in adjustment among youth has opened the door to new questions. First, these two studies operationalized asynchrony as a difference in the progression of two body parts. For girls, both studies focused on the discrepancy in the development of breast and body hair. For boys, Stumper et al. [19] examined the difference between voice change and body hair growth (as well as facial hair and body hair growth). However, these body parts are not the only facets of growth involved in pubertal maturation. One may apply a more multidimensional approach and ask whether asynchrony in the coordination among multiple puberty-related body parts has a similar impact. To do so requires a step away from a simple subtraction method that relies on a difference score between two items. Instead, it is necessary to apply a computational strategy that can incorporate variability across multiple items. 

Second, these two studies demonstrated the effect of asynchrony on depressive symptoms; however, the impact of asynchrony on other domains of psychosocial functioning remains unknown. As noted earlier, the salient domain that may be directly affected by asynchronous physical appearance is social relationships, specifically peer victimization. Because pubertal asynchrony creates a physical appearance in which body parts grow disproportionately, one’s overall appearance may appear gawky and awkward at times. As physical appearance is an important correlate of peer relationships among youth in early adolescence [9,10], we speculate that asynchronously developing youth might be subject to stigmatization and victimization. 

Finally, and most notably, the possible sex differences in the association between pubertal asynchrony and peer victimization require special attention. Because puberty is a highly sex-specific process and the growth of each body part has a different social valence for boys and girls [20], the effect of asynchrony may differ by sex. In fact, initial evidence suggests that the association between pubertal asynchrony on depressive symptoms differed by child sex, with pubertal asynchrony being a risk for girls but a protective factor for boys [19]. The sex-specific prediction is particularly plausible in predicting peer victimization. For instance, research on physical appearance and peer harassment indicate that height is a delicate issue for girls; being short is a risk factor for being bullied among both adolescent boys and girls [21,22], but girls who perceive themselves to be too tall are also likely than other girls to report being teased about their appearance [23]. For boys, on the other hand, being tall may be a positive attribute that conveys social dominance [24]. Similarly, other visible signs of puberty, such as acne, could send unwanted social signals, especially for girls based on the social norms and beauty ideals that are culturally prescribed for females [25]. Therefore, a careful sex-specific examination of the social implications associated with each body part is paramount. To our knowledge, this investigation is the first effort to examine pubertal asynchrony and peer victimization in both boys and girls with special attention to the coordination of multiple (i.e., more than two) body parts that are changing simultaneously but semi-independently during the pubertal transition.

### 1.3. Present Study

The overarching aim of this study is to offer a direct response to Mendle’s [1] call for more research on puberty synchrony and psychosocial functioning. First, currently, there is a disconnect between theoretical and empirical work on pubertal synchrony and its psychosocial impact. Building on existing work [18,19], this study evaluated associations between pubertal asynchrony and peer victimization at age 11, a time at which youth are sensitive to peer victimization [10]. The following hypotheses were proposed: First, we expected that pubertal asynchrony would place youth at an increased risk for peer victimization, especially for girls. Second, we speculated exploratorily that some visible signs of pubertal changes, such as height (or sense of becoming “tall and big”) and skin change, would play an important role in explaining the sex-specific effect of pubertal asynchrony. 

Several unique features of this study are worth mentioning. Importantly, we offer a novel approach to compute pubertal asynchrony scores. The few studies that have examined puberty synchrony solely focused on the mismatch between two body parts and considered asynchrony in a categorical approach. In this study, we offer an alternative approach for the simultaneous capture of the variability in multiple body changes (i.e., breast development, growth spurt, body hair growth, menarche, and skin change for girls and growth spurt, voice change, body hair growth, facial hair appearance, and skin change for boys). Another benefit of this approach is its ability to compute a continuous scale of asynchrony that is sensitive to subtle individual differences. Additionally, aligned with Stumper et al. [19], this study included both boys and girls to evaluate sex-specific effects of pubertal asynchrony. We also supplemented with a person-oriented analytical technique (i.e., cluster analysis) to capture how the physical appearance of asynchronously maturing youth looks like at age 11. The illustration of asynchronously developing body type would provide an insight into why asynchronous look would be associated with peer victimization in boys and girls. 

## 2. Methods

### 2.1. Participants

This study utilizes data from the Early Growth and Development Study (EGDS) [26]. The EGDS is a prospective, longitudinal adoption study that has followed adoptees and their biological and adoptive parents (*n* = 561) since the adoptees were in infancy. All EGDS adoptees were adopted in the first few days after birth (median age of the child at adoption placement = 6 days, *SD* = 13.19 days, range = 0 to 91 days). The EGDS sample was recruited from 45 agencies in 15 states across the U.S., reflecting the full range of U.S. adoption agencies, including public, private, open, closed, religious, and secular adoptions. Families were considered eligible for the study if they met the following criteria: (a) the adoption placement was domestic within the U.S.; (b) voluntary adoption placement occurred within three months after the birth of the child; (c) the adopted infant was biologically unrelated to the adoptive family; (d) no major medical conditions were present at birth; and (e) the birth and adoptive parents had English proficiency at the eighth-grade level. The protocols were approved by the institutional review board at the University of Oregon (Protocol # 08082016.007, approved on 8/11/2021) and other data collection sites. Detailed information regarding recruitment and sample characteristics is provided in Leve et al. [26].

The sample for this investigation consisted of 413 adoptees (237 boys, 176 girls) and their adoptive parents who participated in the age 11 assessment. The race/ethnicity breakdown of the adopted children in our analytical sample was as follows: 55.4% White, 12.3% Black/African American, 22.0% multiracial, 9.2% Hispanic/Latino, and 1% other. Adoptive parents were typically college-educated and from middle- to upper-class families, with more than half of the households reporting incomes higher than $100,000 per year. Ninety-two percent of adoptive mothers and 91.9% of adoptive fathers were Caucasian. Moreover, 81.4% of adoptive mothers and 90.6% of adoptive fathers were married at the time of the age 11 assessment.

### 2.2. Measures

*Pubertal Asynchrony and Status/Timing*. At the age 11 assessment, youth responded to the Pubertal Development Scale (PDS [27]) to describe the puberty-related changes in their bodies. Using the four-point scale (1 = *no change* 4 = *completed change*), boys rated their growth on facial hair, growth spurt, skin change, voice change, and body hair (a = 0.61). Girls described their maturation in terms of growth spurt, skin change, body hair, and breast development using the same four-point scale boys used, and they also recorded the occurrence of menarche in a yes/no choice (a = 0.61). Following the conventional scaling protocol, a yes response was coded as 4, and a no response was coded as 1 to harmonize the response options with the rest of the PDS items.

To capture the variability in the maturation of body parts, we created a *pubertal asynchrony* score by computing the standard deviations of five items for each respondent. Higher scores for this variable indicate a greater variation in the status of body changes, i.e., greater asynchrony. Therefore, a score of 0 on the asynchrony scale means that all the body parts assessed by the PDS were at the same status and were progressing synchronously.

As noted earlier, pubertal asynchrony is somewhat dependent on pubertal status; pubertal asynchrony is minimal at the beginning (i.e., floor effect) and toward the end of the pubertal transition (i.e., ceiling effect). To capture the context in which the ascertained asynchrony occurred, we also computed the overall *pubertal status* for each youth. We adopted the coding system developed by Shirtcliff and colleagues [28], which aligns the PDS scores to the Tanner stages of pubertal development, where 1 indicates *no development* and 5 indicates *adult development.* This coding system is able to separately capture the adrenal and gonadal hormonal signals of physical development based on various signs of physical maturation that are differently related to adrenal and gonadal signals [28]. More specifically, gonadal hormone development is associated with growth spurt, breast development, and menarche in girls and growth spurt, deepening of the voice, and facial hair growth in boys. Adrenal hormones, on the other hand, are associated with pubic/body hair growth and skin change in both sexes. A total pubertal status score was calculated by averaging the adrenal and gonadal scores of both boys and girls. The pubertal status score was included in the subsequent models as a covariate. It is noteworthy that the operationalization of pubertal status in this study was the same as that of pubertal timing, as this study uses a cohort sequential design according to which pubertal status and timing are mathematically synonymous.

Importantly, readers are reminded that our measure of puberty is based on youth’s self-perceptions of physical growth, which is correlated but not equivalent to objective physical development [29]. Thus, it is important to keep in mind that our measures of puberty asynchrony and status are based on how youth interpret their physical development at the time and in contexts that were relevant to them. 

*Peer Victimization***.** Youth responded to the Multidimensional Peer Victimization Scale [30]. This 16-item scale assesses four types of victimization school children may experience, including physical victimization (a = 0.79), social manipulation (a = 0.72), verbal harassment (a = 0.78), and attacks on property (a = 0.78). Youth were asked about the frequency of these harassment incidents during the last school year. The responses were rated with a three-point scale (0 = *not at all,* 1 = *once*, and 2 = *more than once*), with higher scores indicating more instances of being bullied. Sample items include the following: “How often during the last school year has another child punched you?” “How often during the last school year has another child tried to break something of yours?” Previous work has shown that this scale corresponds to global self-identification as a bullying victim [30]. In this sample, 81.2 % of youth reported at least one peer victimization incident during the last school year. More specifically, 40.3% of youth reported at least one incident of physical victimization, 64.7% reported an incident of social victimization, 66.6% reported verbal victimization, and 51.9% reported property attacks. 

The four subscales (physical victimization, social manipulation, verbal harassment, and attacks on property) were significantly correlated with each other (*r*-values ranging between 0.55 and 0.62, which are consistent with previous work (see [31] for a review). We created a composite peer victimization score by standardizing and adding the subscales together. The internal consistency estimate for the composite score was 0.90.

*Covariates.* Several covariates were considered, including adoptive parents’ marital status and household income, youth race/ethnicity, body mass index (BMI), and social skills. We chose these variables as potential covariates because (1) these sociodemographic variables are known to be associated with peer victimization [32]; (2) race/ethnicity is associated with puberty [33,34] and peer victimization [35]; (3) higher BMI, which is correlated with pubertal maturation [34,36], is also a risk factor for peer victimization [37]; and (4) social skills (or lack thereof) are a well-established correlate of peer victimization [38]. 

Adoptive parents reported their marital status on a seven-point scale (1 = single/never married, 2 = single/widowed, 3 = married, 4 = married but separated, 5 = divorced/not married, 6 = remarried, and 7 = living in a committed, married like relationship). A composite measure of marital status was created by using mothers’ reports of marital status where fathers’ reports replaced the missing values. The marital status variable was further recoded for simplification with 0 = married, 1= others. 

Adoptive parents reported on their yearly *household income* when youth were 11 years old using a 10-point scale where 0 = *less than $15,000* a year to 10 = *more than $300,000 a year*. We used the primary caregivers’ (mostly mothers) report of income to represent both parents’ report of house income. Missing values of mothers’ reports were replaced by existing values from fathers’ reports.

Youth *race/ethnicity* was coded dichotomously with 0 = *another race/ethnicity* and 1 = *Black*. The coding reflects our plan to test the effect of being Black because Black girls are known to have puberty earlier, on average [33]. 

Youth *BMI* was also used as a covariate in this study. Data on children’s height and weight at age 9 were gathered from their medical records from their physicians and parental reports. Because the timing of doctor visits and parental reports varied across families, there were potentially multiple records of physical measurements for each family. For those cases that had multiple assessments, we calculated multiple BMI scores from weight and height for each assessment occasion, which were then averaged into a final measure of BMI. BMI and associated weight status are known to be stable in middle childhood and early adolescence [39,40] 

Lastly, youth’s *social skills* were ascertained using the social skills subscale of the Social Skills Improvement System [41]. Adoptive mothers and fathers independently reported how often their children exhibited certain social skills (0 = *never*, 1 = *sometimes*, 2 = *very often,* 3 *=almost always).* Higher scores indicate better social skills. Mothers’ (a = 0.95) and fathers’ (a = 0.95) reports were highly correlated *(r* = 0.56, *p* < 0.001), and thus, a composite score was created by averaging these ratings. 

### 2.3. Missing Data

The evaluation of the missingness pattern indicated that 0.24% to 43% of the data used in this study were missing depending on the variables, with BMI having the most frequently missed data. We ran Little’s missing at completely random (MCAR) test separately by sex (because some of the puberty items were sex-specific and missing for a valid reason). Results supported that the data were MCAR (for boys, $\chi_{\left(125\right)}^{2}$ = 124.19, *p* = 0.263; for girls, $\chi_{\left(114\right)}^{2}$ = 123.65, *p* = 0.253). Therefore, we proceeded with full information maximum likelihood (FIML) to treat the missingness in the subsequent analyses.

### 2.4. Analytic Strategy

The analytic steps were planned as follows. First, descriptive statistics were presented. Second, to test the effect of puberty asynchrony on peer victimization, we ran a multigroup path analysis by child sex, controlling for pubertal status, social skills, BMI, and parents’ sociodemographic variables. The path analysis was conducted using Mplus 8 [42], which uses FIML to estimate model parameters in the presence of incomplete data [43]. To test sex differences in the association between pubertal asynchrony and peer victimization, we applied a multigroup path analysis framework. In this approach, two nested models were compared by the evaluation of fit statistics: a model that freely estimated a specific path (i.e., from asynchrony to peer victimization) for boys and girls (unconstrained) vs. a more parsimonious model where the coefficients of the path were set equal across the two sexes (constrained). Differences between boys and girls were indicated by significant Chi-square difference tests for the constrained versus unconstrained models. To gain further insights into the physical appearance of asynchronous body in boys and girls, we conducted a series of K-means cluster analyses to identify distinct groups of youth with different patterns of (a)synchrony in body parts.

## 3. Results

### 3.1. Descriptive Statistics

Table 1 shows the correlation among puberty-related body changes by sex. Significant positive correlations among body changes were expected and observed, but some body changes were not correlated with each other (*r* values ranging from 0.09 to 0.36 for boys and −0.04 to 0.53 for girls). The variation in correlations suggests the presence of asynchrony among body parts at age 11. In particular, growth spurt was not significantly related to skin changes and menarche in girls and was not associated with facial hair growth in boys. This pattern of associations suggests that at age 11, growth spurt may be one puberty-related body change that may contribute to asynchrony. 

Table 2 provides the frequency distribution of pubertal status by sex. As expected from the age of the sample, many youths were at the beginning of puberty, but many believed that they had grown and were growing. 

Bivariate correlations among study variables along with the corresponding means and standard deviations are presented in Table 3 and Table 4, respectively. As shown in Table 3, the results of bivariate correlations show that the direction of the association between pubertal asynchrony and peer victimization differed by sex. In girls, asynchronous development was *positively* associated with peer victimization *(r* = 0.16, *p* = 0.042). However, in boys, puberty asynchrony was *negatively* associated with peer victimization, although the association was only marginally significant (*r* = −0.12, *p* = 0.074). Pubertal status was significantly associated with peer victimization for boys (*r* = 0.16, *p* = 0.021), but not for girls (*r* = −0.03, *p =* 0.734). BMI was significantly associated with pubertal status in boys (*r* = 0.25, *p* = 0.005), but not in girls (*r* = 0.11, *p* = 0.293). Moreover, BMI was not associated with asynchrony in boys (*r* = 0.13, *p* = 0.145), but was marginally associated with asynchrony in girls (*r* = 0.19, *p* = 0.070). Social skills and race/ethnicity, as well as parents’ household income and marital status, were not correlated with pubertal asynchrony or peer victimization for either boys or girls, except for one marginal association between household income and peer victimization in girls (*r* = −0.16, *p* =0.099).

### 3.2. Pubertal Asynchrony and Peer Victimization: Path Analysis

As the descriptive results showed that pubertal asynchrony operates differently depending on the sex of the youth, subsequent analyses used a multigroup path model. First, an unconstrained model was used to evaluate the patterns of asynchrony effects for each sex globally. This unconstrained path model was just-identified. Thus, we constrained one of the paths (i.e., from race/ethnicity to peer victimization) to zero to save the degrees of freedom that were necessary to compute the fit indices. We chose the path from race/ethnicity to peer victimization to set at zero because it was minimally correlated with the endogenous variable. All independent variables were mean-centered, except for pubertal status, which was centered at the lowest possible stage of pubertal development (i.e., Stage 1) for ease of interpretation. Of note, the asynchrony score that we created was suitable for capturing the variability of multiple pubertal changes, but it did not convey information about the pubertal stage at which the variability occurred. To understand this “anchor” question, we added an interaction term between pubertal asynchrony and pubertal status to the models described above. None of the interactions were significant, and thus, this term was omitted from the subsequent analyses.

**Figure 1:**
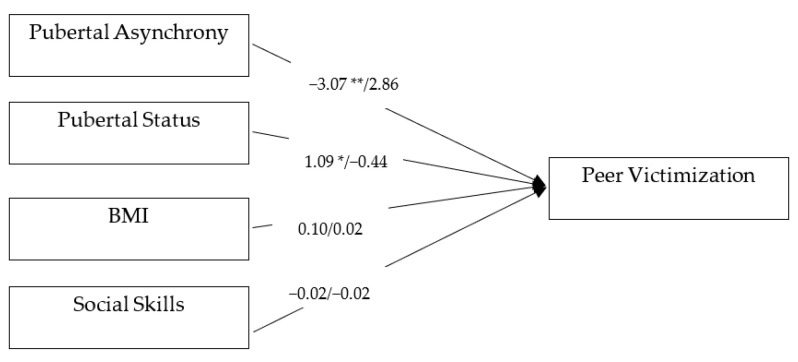
The effects of pubertal asynchrony and pubertal status on adolescents’ peer victimization at age 11. Note. * *p* ≤ 0.05. ** *p* ≤ 0.01. Girls are in the right side of the slash. Covariates including race/ethnicity, parental marital status, and income were included in the model but not shown here for simplicity. Figure reports unstandardized coefficients. Fit indices: χ^2^(_2_) = 0.61, *p* = 0.73, CFI = 1.00, TLI = 1.00 RMSEA = 0.00, 90 % CI for RMSEA = [0.00, 0.13].

The results for the unconstrained two-group model are presented in Figure 1. Model fit indicated good fit of model to data, χ^2^_(2)_ = 0.61, *p* = 0.736 RMSEA = 0.00, 90 % CI for RMSEA = [0.00, 0.13, CFI = 1.00 and TLI =1.00]. As shown in Figure 1, pubertal asynchrony was negatively associated with peer victimization in boys (*b* = −3.07, *p =* 0.008, 95% CI [−5.34, −0.79]), indicating that pubertal asynchrony puts boys at a lower risk for peer victimization. Boys’ pubertal status was also significantly associated with peer victimization (*b* = 1.09, *p* = 0.020, 95% CI [0.15, 2.02]). On the other hand, girls’ pubertal asynchrony was positively associated with peer victimization (*b* = 2.86, *p* = 0.021, 95% CI [0.42, 5.29]), such that girls whose body parts grew at uncoordinated paces experienced more peer victimization than girls who developed synchronously. However, pubertal status was not associated with victimization in girls (*b* = −0.44, *p* = 0.265, 95% CI [−1.22, 0.23]). 

Next, to formally conduct a test of sex differences in pubertal asynchrony effects, the fully unconstrained model (mentioned above) was compared to a more parsimonious model with a constraint of the equal coefficient being imposed on the asynchrony-victimization path for both boys and girls. A poor model fit was observed for the constrained model: $\chi_{\left(3\right)}^{2}$ = 12.45 *p* = 0.006, CFI = 0.00, TLI = 0.00, RMSEA = 0.17, 90% CI for RMSEA = [0.08, 0.27]. The comparison between the freely estimated and constrained models indicated a significant change in the Chi-square value ($∆\chi_{\left(1\right)\;}^{2}$ = 11.84, *p* < 0.001), suggesting that the effect of asynchrony on victimization was different between the sexes. Altogether, these findings indicate that pubertal asynchrony was associated with peer victimization above and beyond the effect of pubertal status, social skills, BMI, and other sociodemographic characteristics, but the pattern of associations was significantly different for boys and girls. Asynchronous boys experienced fewer instances of peer victimization than their more synchronous counterparts. However, this pattern was the opposite for girls; asynchronous girls were more likely to be a target of peer victimization than their synchronous counterparts.

### 3.3. Asynchronously Developing Body: Cluster Analysis

The aforementioned results raise additional questions. How does an asynchronous body look like in girls and boys at age 11? Which body part(s) drives the asynchrony scores? Answers to these questions may provide illustrative ideas as to why asynchronous bodies serve as a protection against peer victimization for boys and a risk for girls. To address these questions, we conducted a *K*-means cluster analysis using five items from the PDS. The challenge of cluster analysis, as with other techniques, such as factor analysis, is the selection of the number of clusters. The selection decision should be based on the empirical examination of several different cluster solutions while attending to theoretical interpretability [44]. We tested between two and five cluster models for boys and girls separately. The comparison of Calinski-Harabasz (CH) statistics [45,46] showed that the two-cluster model has the maximum CH value for both boys and girls (75.79 and 113.27, respectively). However, the interpretation of the two-cluster solution suggested that the two identified clusters were distinguished by the levels of physical maturity, i.e., one group at the advanced pubertal stage and the other at the less mature stage. As this distinction is not conceptually meaningful for the purpose of the study, we proceeded with the three-cluster solution, which is the second-highest CH value for both boys and girls (CH = 70.30 and 100.82, respectively). The three-cluster solution revealed a third group, namely, asynchronous youth, in addition to the two groups mentioned earlier (i.e., the synchronously mature and immature youth). We also examined the four- and five-cluster solutions, but the CH values resulted in smaller values.

**Figure 2:**
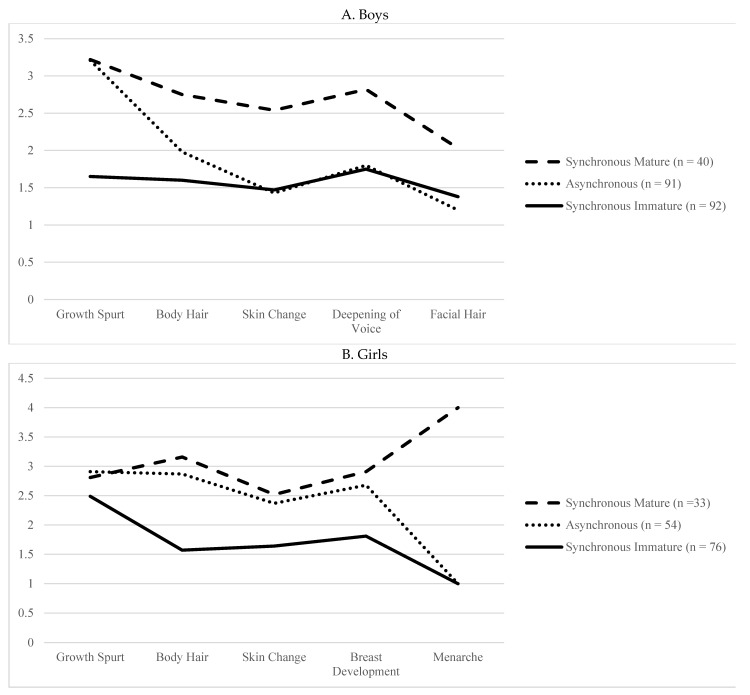
Average maturation levels by puberty-related body changes for boys and girls.

The closer examination of the three-cluster solutions for boys indicated that the largest two groups were synchronous immature boys (*n* = 92) and asynchronous boys (*n* = 91). The group of synchronous mature boys (*n* = 40) formed the smallest cluster. For girls, we identified groups of girls whose body parts were synchronously immature (*n* = 76), synchronously mature (*n* = 33), and asynchronously developing (*n* = 54). The profile means of the three clusters for each sex are presented in Figure 2. To statically test the differences in the pubertal characteristics among the three groups, we conducted a multivariate analysis of variance (MANOVA) of the five pubertal items separately for boys and girls. 

*Boys*. As expected, there were significant differences in the levels of growth based on boys’ cluster membership, *F* (10, 416) = 81.32, *p* < 0.001, Wilk’s Λ = 0.11, partial η^2^ = 0.66.

Post hoc Tukey comparisons indicated that compared to the synchronous-mature group, the asynchronous group experienced less body hair growth (*HSD* = −0.78, *p* < 0.001), fewer changes in skin (*HSD* = −1.11, *p* < 0.001), less voice change (*HSD* = −1.00, *p* < 0.001), and less facial hair growth (*HSD* = −0.88, *p* < 0.001), but they were similarly advanced in terms of growth spurt (*HSD* = 0.05, *p =* 0.605). 

Compared to boys in the synchronous-immature group, the asynchronous boys were more advanced in terms of growth spurt (*HSD* = 1.65, *p* < 0.001) and body hair growth (*HSD* = 0.37, *p* < 0.001), and were less advanced in terms of facial hair growth (*HSD* = −0.17, *p* = 0.041). However, no group differences were observed in skin change (*HSD* = 0.03, *p* = 0.757) or voice change (*HSD* = 0.05, *p* = 0.630).

*Girls.* Likewise, significant differences in the levels of growth based on cluster membership were observed for girls, *F* (8, 304) = 28.18, *p* < 0.001, Wilk’s Λ = 0.33, partial η^2^ = 0.42. Post hoc Tukey comparisons indicated that girls in the synchronous-mature and asynchronous groups were similarly advanced in terms of growth spurt (*HSD* = 0.09, *p* = 0.593), skin change (*HSD* = −0.12, *p* = 0.471), and breast development (*HSD* = −0.23, *p* = 0.092); however, asynchronous girls were less mature than synchronous-mature girls in terms of body hair (*HSD* = −0.29, *p* = 0.037). Regarding menarche, none of the asynchronous girls has reached menarche while all synchronous-mature girls had, $\chi^{2}$(2, *n* = 162) = 162, *p* < 0.001.

The comparison between asynchronous girls and synchronous-immature girls showed that asynchronous girls were more physically advanced in all domains (*HSD* = 0.41, for growth spurt, 1.29 for body hair growth, 0.73 for skin change, and 0.87 for breast development, all at *p* < 0.001) except menarche; none of the girls in either asynchronous or synchronous-immature group had reached menarche. 

## 4. Discussion

The bodies of youth may appear gawky and unbalanced during the pubertal transition because each body part that changes during this phase of life develops semi-independently, creating asynchrony in the body. The overarching aim of this study was to examine the potential social impact of pubertal asynchrony on peer victimization for both boys and girls. Our findings demonstrated that pubertal asynchrony is a risk factor for peer victimization for girls but serves as a protective factor for boys. Asynchronously developing bodies look differently for boys and girls. Asynchronous boys had a tall but otherwise immature look, while asynchronous girls had a look of outwardly mature but covertly immature. 

### 4.1. Pubertal Asynchrony: A Protective Factor for Boys but A Risk Factor for Girls

Mendle [1] proposed two competing hypotheses about pubertal asynchrony. One hypothesis purports that pubertal asynchrony is a risk, while a competing hypothesis suggests that pubertal asynchrony should serve a protective function. Our findings partially support both hypotheses: pubertal asynchrony was found to be a protective factor for boys but a risk factor for girls. 

Our results indicated that asynchronous boys experienced fewer incidents of peer victimization than their synchronous peers. This beneficial effect of pubertal asynchrony was observed above and beyond the effects of well-known correlates of peer victimization and puberty. The observed benefit of pubertal asynchrony in boys is consistent with the finding by Stumper et al. [19].

According to results from our cluster analysis, boys’ asynchrony was primarily driven by a growth spurt. The group of asynchronous boys was characterized by experiences of growth spurt without signs of maturation in other body parts. Although more research is needed to identify the mechanisms, we speculate that asynchronously developing boys who are becoming taller may enjoy social advantage while dodging risks associated with the look of older boys. From the adult literature, evidence suggests that tall individuals, especially men, may enjoy social advantages, such as prestige and leadership because tall height is perceived as a sign of social dominance [24]. Even children as young as preschoolers associate tall stature with not only physical strength but also social prowess [47]. The appearance of an overall “mature look” is a likely risk factor for peer victimization [10], as shown in the positive association between pubertal status and peer victimization in boys. However, in the case of asynchronous boys, the social advantage associated with tall height (or becoming tall) without having an overall mature look may protect them from peer harassment. 

While growth spurt appears to play an essential role in boys’ peer experiences, readers are reminded that because we used the youth reported PDS, our findings are based solely on a subjective evaluation of boys’ growth spurt. This pattern may explain why more than half of boys reported positively to the presence of growth spurt as early as age 11. Because growth spurt usually starts at the later phase of boys’ pubertal maturation, many boys may not have experienced actual growth spurt had we conducted a physical exam. However, because we assessed self-perceptions of pubertal maturation, we see much greater variability (see Table 2) in their responses, with many boys reporting that they were growing tall. Joining Mendle’s [1,29] call, we argue that the subjective experience and anticipation of becoming “big and tall” is an important correlate of social functioning for boys. 

The experience of pubertal asynchrony among girls was different from that of boys. Pubertal asynchrony puts girls at risk of peer victimization above and beyond pubertal status and other covariates. The finding is consistent with prior work showing the positive link between pubertal asynchrony and maladjustment, such as depression, in girls (Stumper et al., 2020; Thompson et al., 2016). In explaining the association between pubertal asynchrony and psychopathology, Mendle [1] speculated that asynchronous youth might experience heightened confusion and uncertainty because each body part grows unpredictably at its own pace. Our findings add a possible expansion of this prediction; the undesirable impact of pubertal asynchrony may also involve social pathways whereby uncoordinated physical appearances seen in asynchronous bodies may invite psychological and physical victimization from peers, which eventually puts girls at risk for psychological morbidity.

What does an asynchronous girl’s body look like? The results from our cluster analysis indicated that the visible signs of body changes (i.e., breast development, growth spurt, and skin change) did not differentiate the asynchronous vs. synchronous mature bodies. However, compared to synchronously maturing girls, asynchronous girls were less developed in terms of the covert signs (i.e., pubic hair and menarche). Interestingly, the path analysis revealed that advanced pubertal status/timing was not a significant risk for peer victimization for girls. These findings suggest that it is not the level of advanced maturity but rather the asynchrony between the overt and covert body parts that mattered for peer victimization. Why would an asynchronous body that looks matured outwardly, but immature covertly invite peer harassment? More research is needed, but we speculate that the confusion and negative anticipations about physical changes held by girls who look mature outside but yet to have menarche may account for some of this association. Evidence illustrates that compared to post-menarcheal girls, pre-menarcheal girls tend to hold more distorted expectations about menstruation, including more anticipated menstruation symptoms and distress [48]. A qualitative work echoes similar findings, showing that premenarcheal girls feel ashamed and worried about menarche, especially when they are not well prepared for menstruation [49]. These findings suggest that when girls are yet to have firsthand experiences of menses, they develop anticipation shaped by ambiguous cultural beliefs and misinformation about menstruation, which may inflate negative evaluations and confusion about physical selves [48]. Further, poor body esteem is a known correlate of peer victimization [23,50]. Thus, as Mendle [1] suggested, asynchronous development may lead to confusion and inflated negative expectations about maturing bodies, disrupting asynchronously developing girls’ mental and social worlds.

### 4.2. Strengths, Limitations, and Future Directions

The credibility of the aforementioned findings is bolstered by several unique features of this investigation. First, to our knowledge, this is the first study to evaluate the role of pubertal asynchrony in both boys’ and girls’ social development. Second, this study joins the effort put forth by Stumper et al. [19] in testing the sex-specific effect of pubertal asynchrony. Third, we applied a unique computational approach to capture the variability in the growth of multiple body parts. 

However, the findings reported here need to be viewed alongside several limitations. First and most important, the pubertal asynchrony scores used in this study were derived from youth’s self-report. Although youth-reported PDS correlates with objective indices of puberty [28] and is good at capturing developmental progression in pubertal maturation [51], self-perceived pubertal maturation is unique in its own right. For example, the physical examination of breast development by trained examiners may differentiate breast tissue and adipose tissue, but for youth, this distinction may be trivial for their experiences [1]. Thus, the observed asynchrony effect seen in this study is asynchrony driven by a perceived sense of physical growth. Second, the effect of pubertal asynchrony observed here may be specific to 11-year-olds. For example, asynchrony was heavily driven by perceived growth height for boys, but different body parts would likely be the driver of asynchrony at different ages. One fruitful avenue for future research is to uncover different types of asynchronies (e.g., boys with unchanged voice but otherwise look mature, girls with acne but otherwise look immature), and how their navigation of the peer world may differ from the asynchronous groups we identified in this study. Third, it is important to note that PDS does not include any item regarding the size of testes, which is usually the first change in boys [4]. As this study was conducted when boys were still in early adolescence, the omission of this item limits the study’s ability to detect early signs of pubertal change in boys. Fourth, the reliability coefficients of PDS were 0.61. However, it is important to note that psychometrically, it is expected to have lower-than-usual alpha levels in the context of assessing pubertal asynchrony because each item in the measure seeks to capture the level of maturation in a specific body part which is influenced by semi-independent hormonal processes, creating asynchrony [29]. Fifth, the evaluation of the association between pubertal asynchrony and peer victimization was based on a cross-sectional design, which limits the delineation of a timeline of events. Nevertheless, a cross-sectional design of this kind may be conceptually suited for this study because from youth’s subjective experience, it is logical to suppose the concurrent relations between how one’s body parts are coordinated and how one is treated by peers rather than how the coordination of body parts is related to peer victimization in the distant future. However, readers are reminded that longitudinal studies designed to disentangle the direction of effects are needed because puberty and peer victimization may be more entangled than assumed. For instance, powerful social stressors, such as peer harassment, can disturb the stress response system that is also implicated in the neuroendocrine mechanisms underlying pubertal maturation, such as the hypothalamus–pituitary–adrenocortical axis and hypothalamus–pituitary–gonadal axis [52]. Therefore, future research is encouraged to adopt a longitudinal design that delineates the sequence of events. Sixth, the assessment of peer victimization relied solely on self-reports by youth, which are known to diverge from other-reported victimization [53]. Our design whereby both pubertal asynchrony and peer victimization were reported by youth makes it impossible to rule out any third variable effects, including shared method variance issues. For instance, youth who have poor body image, or more generally low self-esteem, may perceive their physical appearance gawky and their peer relationships struggling. Seventh, due to the limited scope of this study, we did not evaluate how different forms of victimization (e.g., sexual harassment, physical victimization) are uniquely associated with pubertal asynchrony. Future work may conduct a close investigation on the forms of victimization. Finally, this report represents one of the few initial efforts to evaluate the role of pubertal asynchrony on youth’s social development, and thus, replication studies are needed.

## 5. Conclusions

Despite the premise that puberty is a normative (and normal) transition that eventually passes with time, it involves “growing pains” [3] for some youth. In this study, we focused on pubertal asynchrony as a potential contributor to growing pains. The results demonstrated that the effect of pubertal asynchrony on social relationships among youth is highly nuanced, playing a role of risk for some youth and protection for others. These results highlight puberty as a unique transitional time when biology intersects with social context. 

## Figures and Tables

**Table 1:** Bivariate Correlations Among Pubertal Facets in Boys and Girls.

		1		2		3		4		5	6		7	
**1**	Growth Spurt	–		0.22		−0.04		n/a		n/a	0.24	**	0.05	
**2**	Body Hair Growth	0.36	**	–		0.38	**	n/a		n/a	0.53	**	0.44	**
**3**	Skin Changes	0.15	*	0.30	**	–		n/a		n/a	0.39	**	0.26	**
**4**	Deepening of Voice	0.16	*	0.28	**	0.20	**	–		n/a	n/a		n/a	
**5**	Facial Hair Growth	0.09		0.33	**	0.22	**	0.35	**	–	n/a		n/a	
**6**	Breast Development	n/a		n/a		n/a		n/a		n/a	–		0.39	**
**7**	Menarche	n/a		n/a		n/a		n/a		n/a	n/a		–	

*Note:* below diagonal are boys and above diagonal are girls. * *p* ≤ 0.05. ** *p* ≤ 0.01.

**Table 2:** Summary of Developmental Progression by Self-Perceived Tanner Stages.

Tanner Stage	Boys		Girls	
	Frequency	Percentage	Frequency	Percentage
1	49	22%	19	11.7%
2	113	50.7%	73	44.7%
3	49	22%	53	32.6%
4	11	4.9%	16	9.8%
5	1	0.4%	2	1.2%

**Table 3:** Inter-Correlations Between Study Variables.

		1		2		3		4		5	6		7		8	
**1**	Pubertal Asynchrony	–		0.22	**	0.16	*	0.19	^+^	−0.04	0.11		0.10		0.01	
**2**	Pubertal Status	0.33	**	–		−0.03		0.11		−0.09	0.09		0.01		0.11	
**3**	Peer Victimization	−0.12	^+^	0.16	*	–		0.02		−0.14	0.03		0.03		−0.16	^+^
**4**	BMI at Age 9	0.13		0.25	**	0.11		–		0.12	0.15		0.17		0.07	
**5**	Social Skills	−0.05		−0.12		−0.08		0.01		–	−0.09		0.00		0.09	
**6**	Race/Ethnicity	0.09		0.12	^+^	0.12		0.16	^+^	−0.04	–		0.07		−0.08	
**7**	Parents' Marital Status	0.04		0.04		0.06		0.21	*	−0.01	0.11	^+^	–		−0.15	
8	Parents' Household Income	0.01		−0.07		−0.09		−0.08		0.01	0.02		−0.43	**	–	

*Note*: race/ethnicity: 0 = everyone else, 1 = Black. Marital status: 0 = married, 1 = others. Boys’ correlations are below diagonal. ^+^ *p* < 0.10. * *p* ≤ 0.05. ** *p* ≤ 0.01.

**Table 4:** Means and Standard Deviations of Study Variables.

	Boys				Girls			
Variables	Mean	SD	Min	Max	Mean	SD	Min	Max
Pubertal Asynchrony	0.74	0.29	0.00	1.73	0.83	0.26	0.00	1.64
Pubertal Status ^a^	2.37	0.81	1.00	5.00	2.70	0.84	1.00	5.00
Peer Victimization ^b^	0.36	3.56	−3.22	12.39	−0.51	2.87	−3.22	9.40
BMI	17.64	3.31	11.87	30.53	17.20	2.83	12.12	25.39
Social Skills	99.23	12.76	50.00	99.23	99.38	12.84	58.50	127.50

Note. ^a^ Pubertal status is in units of Tanner stages. ^b^ Peer victimization values represent standardized values.

## Data Availability

The datasets generated and/or analyzed during the current study are not publicly available but may be available from the corresponding author on reasonable request.
